# Transoral robotic reconstruction of a posterior palatal defect using an upper arm flap following polymorphous adenocarcinoma resection: a novel minimally invasive approach

**DOI:** 10.1007/s10006-026-01639-0

**Published:** 2026-09-19

**Authors:** Henning Wieker, Juliane Wagner, Jörg Wiltfang, Aydin Gülses, Johannes Spille

**Affiliations:** 1 Department of Oral and Maxillofacial Surgery, Christian-Albrechts-University, UKSH Campus Kiel, Kiel, Germany; 2 Department of Oral and Maxillofacial Surgery, University Hospital of Schleswig- Holstein, Campus Kiel, Arnold-Heller-Straße 3, 24105 Kiel, Germany

**Keywords:** Robotic oral tumor, Minimal invasive, Supermicrosurgery, Polymorphous adenocarcinoma, Minor salivary gland, Neck management

## Abstract

**Background:**

Surgical management of malignant tumors in the orofacial region frequently necessitates extensive resection, resulting in significant functional and aesthetic morbidity. In younger patients, these sequelae carry a substantial psychosocial burden, emphasizing the need for minimally invasive reconstructive strategies that preserve quality of life while maintaining oncological safety.

**Case presentation:**

A 36-year-old female patient presented with a small 1 × 1 cm swelling at the junction of the hard and soft palate on the left side. Incisional biopsy confirmed a malignant minor salivary gland tumor but did not permit definitive subtyping. The final diagnosis of a polymorphous adenocarcinoma (PAC), conventional subtype, was established only on the complete resection specimen. Following oncologically adequate resection, including partial hard palate and tuberosity removal, a 3 × 4 cm defect was reconstructed with a free upper arm flap. Supermicrovascular anastomoses to the superior labial and facial arteries (vessel diameter ~ 0.2 mm) were performed via a transoral approach using the Symani^®^ robotic surgical system with 20× motion scaling. Venous anastomoses to the facial vein were completed end-to-end and end-to-side via the same intraoral access. Selective lymph node sampling of the four radiologically prominent ipsilateral level II lymph nodes through a minimal cervical incision showed no metastases, both on frozen section and on final histopathology (0/4). Final histopathology demonstrated a 15 mm low-grade PAC resected in sano, without lymphovascular invasion, without unequivocal perineural infiltration and without bone invasion, classified as pT1 pN0 (0/4) cM0, R0. Postoperative course was uneventful; functional rehabilitation including speech and swallowing therapy is ongoing.

**Conclusions:**

Robotic-assisted supermicrosurgery using the Symani^®^ system enabled safe transoral free flap reconstruction of a posterior palatal defect with anastomoses at the sub 0.2 mm scale. This approach minimizes donor-site morbidity and demonstrates the feasibility of intraoral robotic supermicrosurgery as a novel reconstructive modality in head and neck oncology.

## Background

Carcinoma of the salivary glands is rare, most frequently arising in the parotid and submandibular glands. Minor salivary gland involvement is even rarer, with the palate being the most common site when minor glands are affected [1]. Minor salivary gland tumors account for approximately 2–4% of all head and neck tumors, and polymorphous adenocarcinoma (PAC), formerly designated polymorphous low-grade adenocarcinoma, is one of the most frequent malignancies at this site [2, 3]. Around 90% of these tumors arise within the oral cavity, most commonly in the hard palate, followed by the buccal mucosa and the retromolar region, whereas extraoral locations account for only about 10%. There is a clear female predilection with a peak incidence in the fifth and sixth decades of life, and the tumors typically present as painless, slow-growing masses covered by non-ulcerated mucosa, ranging between 1 and 4 cm in size [2]. In contrast to high-grade salivary gland malignancies, the conventional variant of PAC follows an indolent course: in a population-based analysis of 460 cases, 70.5% of patients presented with localized disease, distant metastases were rare (4.3%), surgery alone was the predominant treatment (77.9%), an elective neck dissection was performed in only 5.3% of patients, and the 10-year disease-specific survival was 96.4% [3]. An institutional series of 57 patients confirmed this indolent behavior, with 81% of tumors classified as clinical T1–T2, only 5% presenting with a clinically positive neck, and a 10-year disease-specific survival of 94%. Recurrences were uncommon and tended to occur late [4].

Irrespective of the histological subtype, complete surgical resection with clear margins represents the cornerstone of the treatment of malignant minor salivary gland tumors, and margin status is among the strongest predictors of local recurrence [2, 4, 5]. This is of particular clinical relevance because the precise entity frequently cannot be established on an incisional biopsy alone. PAC displays a wide morphological spectrum and may be misinterpreted as pleomorphic adenoma, carcinoma ex pleomorphic adenoma, adenoid cystic carcinoma or adenocarcinoma not otherwise specified, so that immunohistochemistry and, ultimately, the assessment of the complete specimen are required for a definitive classification [2]. Consequently, oncologically adequate resection of the primary tumor must not be delayed while awaiting definitive subtyping, whereas the extent of cervical treatment is entity-, grade- and stage-dependent and can therefore reasonably be deferred until the final histopathological diagnosis is available [6].

Surgical resection of posterior palatal tumors is inherently challenging due to restricted transoral access, proximity to critical neurovascular structures, and the need to restore functional outcomes such as speech and swallowing. Traditional extraoral approaches, including Weber–Ferguson and other facial incisions, provide broad exposure but are associated with significant morbidity, including facial scarring and infraorbital nerve injury, often necessitating prosthetic rehabilitation and adversely affecting quality of life, especially in younger patients [7].

Even when local or regional flaps are used, limitations persist. The pedicled buccal fat flap (PBFF) has been widely utilized for intraoral defect reconstruction, particularly for closure of oroantral and oronasal fistulas, due to its ease of harvest and low donor-site morbidity. However, persistent postoperative complications occur in approximately 31% of PBFF cases, particularly in hard and soft palate defects, and their reach may be limited in extensive posterior palatal defects [8]. Defect-oriented reconstruction algorithms employing buccinator myomucosal flaps — including axial, island, and tunnelized variants — have shown favorable outcomes in speech and swallowing recovery with low donor-site morbidity, but they remain constrained in large posterior maxillary defects [9, 10].

Robotic-assisted microsurgical platforms, such as the Symani^®^ Surgical System, offer an innovative solution in anatomically constrained fields. This system enables tremor filtration, motion scaling, and wristed instrument control, enabling precise microvascular anastomoses in confined spaces [11]. In the present case, we utilized an upper-arm free flap for transoral reconstruction of the defect following PAC resection, with vascular anastomoses to the superior labial and facial artery branches. This approach avoided extraoral incisions, minimized morbidity, and enabled functional restoration, representing a novel strategy for minimally invasive posterior palatal reconstruction. Performing supermicrosurgery with a robot via the oral cavity is new and represents a new possibility in reconstructive surgery.

## Case presentation

**Fig. 1:**
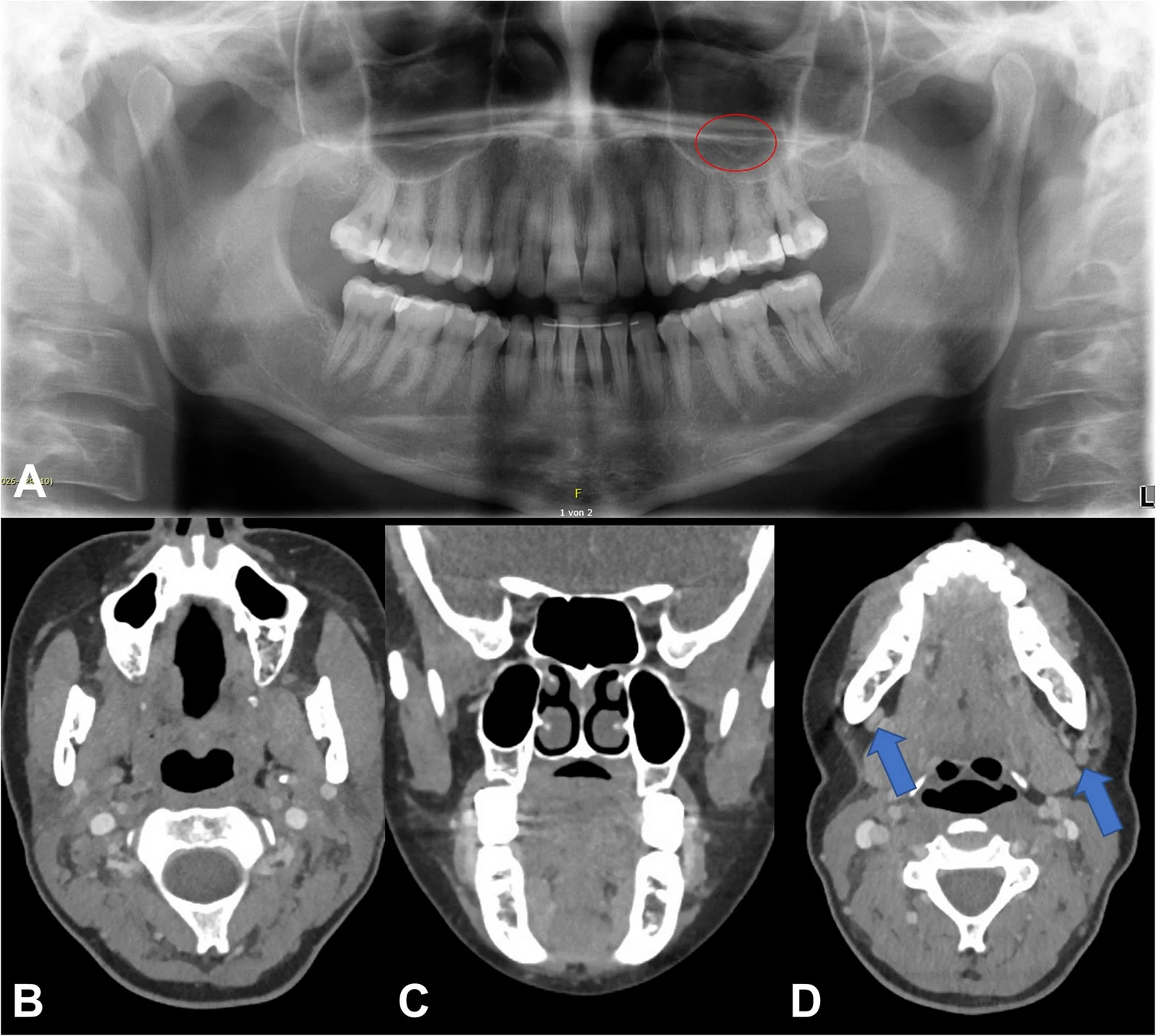
(**A**) Preoperative imaging. On the left side, in the area of the maxillary sinus, a rounded opacity is visible, circled in red. This finding was identified intraorally as the carcinoma. (**B**) and (**C**) show cross-sectional images with no evidence of bone infiltration. In (**D**), the blue arrows point to enlarged lymph nodes

A 36-year-old female patient presented to our oral and maxillofacial surgery department following a routine dental examination. An abnormality was noted on the panoramic radiograph (Fig. 1A). The patient reported a swelling of the palate that had been present for approximately 5 years and had remained unchanged. Clinical examination revealed a tumor size of 1 × 1 cm at the junction of the soft and hard palate on the left side. A biopsy of the affected area was performed on the same day. No relevant medical history, other comorbidities, or prior surgeries were obtained in the anamnesis. A few days later, the pathology report confirmed a malignant minor salivary gland tumor. However, the incisional biopsy did not allow a reliable subtyping of the entity, and a definitive classification was explicitly deferred to the assessment of the complete resection specimen. Staging revealed no further pathological abnormalities in the computed tomography scan of the head, neck (Fig. 1B-D), and thorax. An abdominal and neck ultrasound was also unremarkable.

### The findings of the CT scan of the tumor region and the soft tissues of the neck revealed

Oral cavity/pharynx: The primary tumor cannot be clearly delineated on CT imaging.

Soft tissues of the neck: Prominent cervical lymph nodes—exemplified by a node in level IIa on the rightmeasuring approximately 10 x 6 mm in axial view and a prominent node in level IIa on the leftmeasuring approximately 19 x 6 mm in axial view—show no suspicious morphological features.

**Fig. 2:**
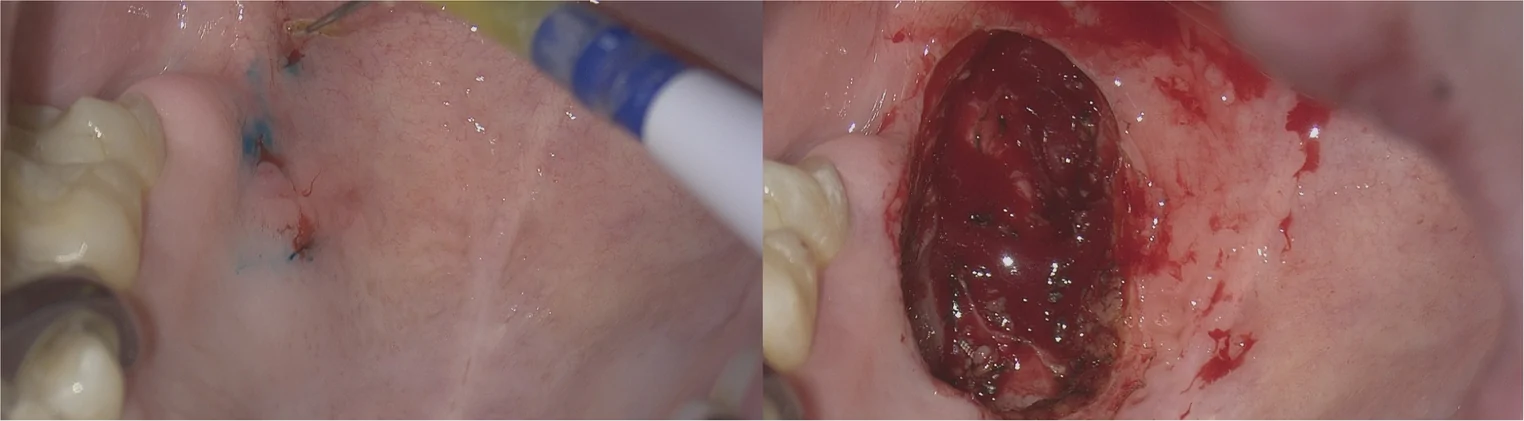
The marked tumor is visible on the left side. A clinical safety margin of 1 cm has already been maintained. The resection is visible on the right side

The patient underwent a transoral resection of the tumor in a first, short operation lasting approximately 1 h (Fig. 2). Histopathological examination revealed a very close margin despite the intraoperative clinical safety margin: the anterior and posterior resection margins were tumor-free, whereas the closest margin was located medially and towards the depth and measured less than 1 mm. We discussed the treatment options in detail with the patient. Due to the patient’s young age, a re-resection with a larger safety margin was indicated. We decided on a radical re-resection to establish a planned clinical resection margin of at least 1 centimeter with partial removal of the hard palate, the maxillary tuberosity, and the second molar. For reconstruction of the defect, we jointly decided on a graft from the upper arm rather than one from the forearm, as the patient uses her arm extensively for work. Furthermore, this avoided a scar on the forearm.

**Fig. 3:**
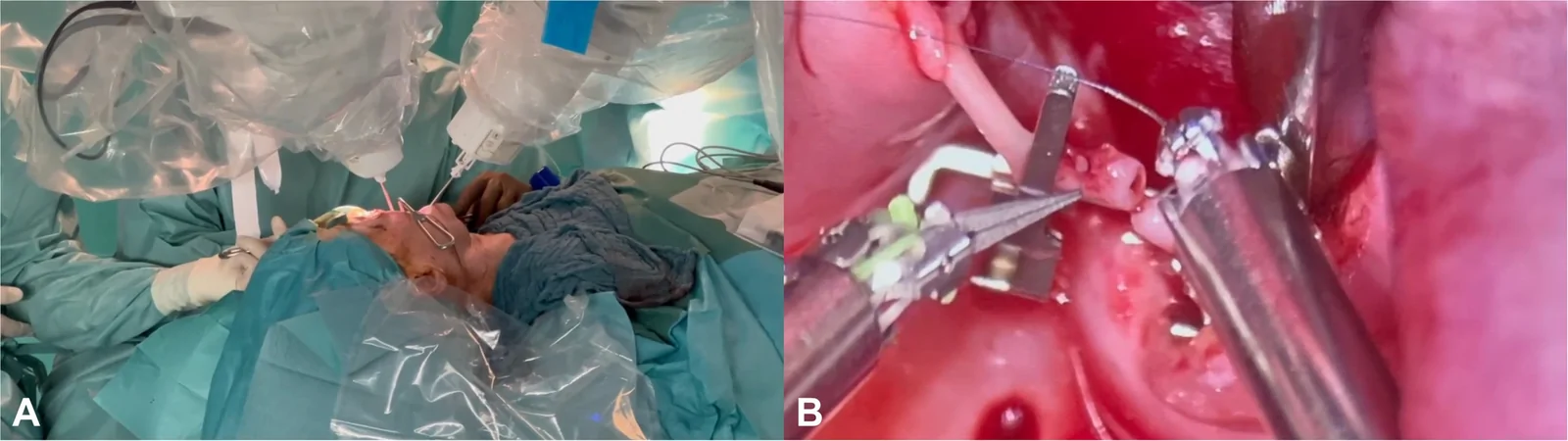
(**A**) shows the surgical setup. The robot’s two arms extend into the oral cavity; the assistant sits beside the arms, handing over suture material and maintaining a dry surgical field. The surgeon does not sit next to the patient but at the console, controlling the robot from there. (**B**) shows the suturing of the anastomosis. Multiple interrupted sutures are placed using the robot’s two instruments

**Fig. 4:**
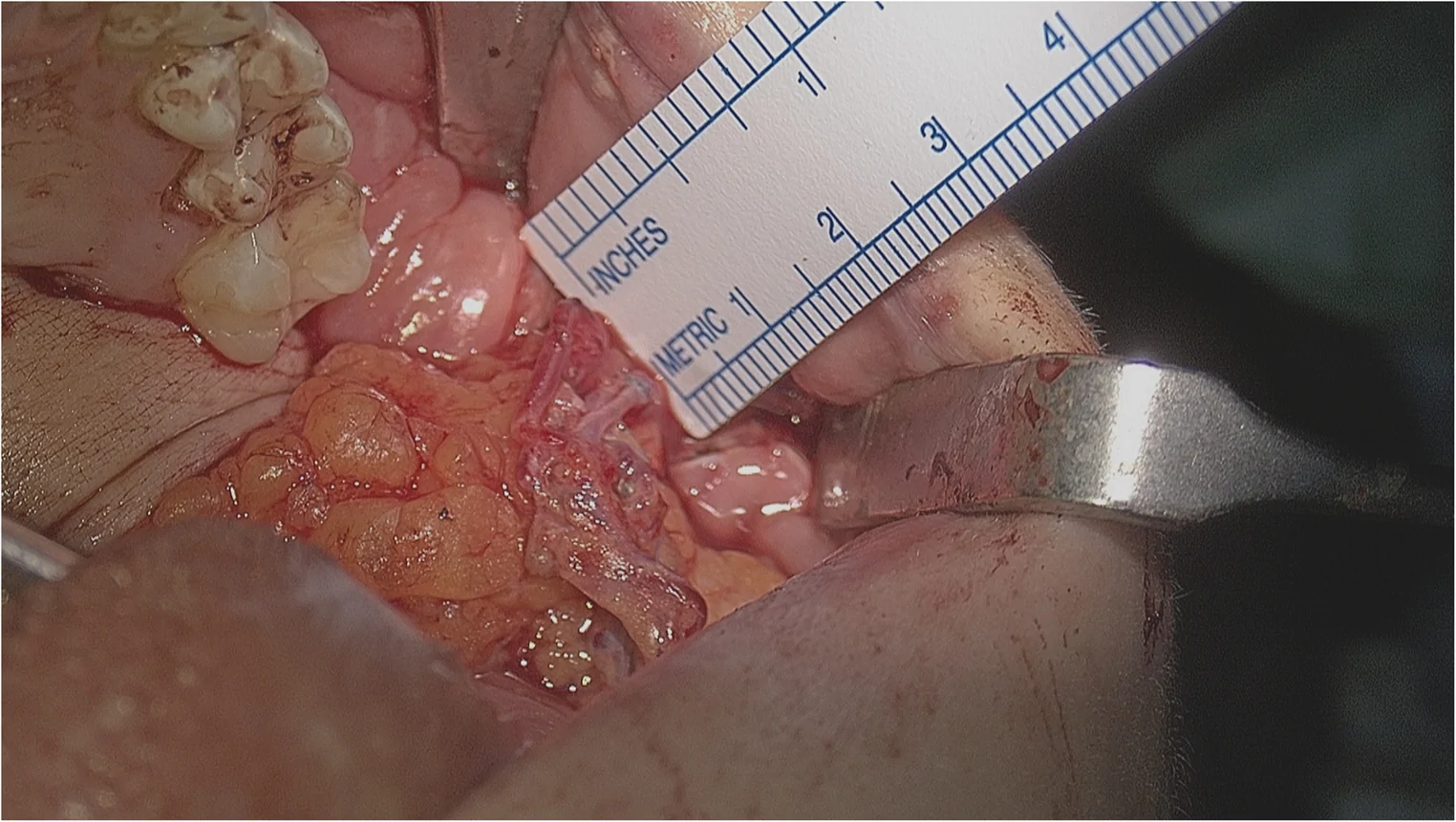
The intraoral arterial anastomoses to the superior labial artery and fine branches of the facial artery. The ruler shows the size of the anastomoses, which have a diameter of approximately 0.2 mm

Intraoperatively, the defect measured approximately 3 × 4 cm and was reconstructed with a free upper arm flap harvested from the left arm (two arteries), with microvascular anastomoses to the superior labial artery and fine branches of the facial artery (Figs. 3 and 4). The veins were also sutured to the facial vein end-to-end and end-to-side. 10 − 0 and 11 − 0 Prolene sutures were used for all anastomoses. The robot was used to perform the surgery with a 20-fold reduction in motion transmission. The anastomoses had a diameter of approximately 0.2 mm. Ischemia time was 123 min.

The robotic system (Symani^®^) facilitated precise dissection and flap inset, allowing complete closure of the defect in a minimally invasive manner. Operative time was nine hours, and intraoperative blood loss was 300 ml. Since both the indication for and the extent of an elective neck dissection depend on histological subtype, grade and tumor stage, a formal neck dissection was deliberately deferred until the definitive histopathological diagnosis of the complete specimen was available. Instead, selective lymph node sampling was performed on the left side, ipsilateral to the primary tumor. The four prominent lymph nodes at level II described in the preoperative CT scan were accessed through a small cervical incision and sent for frozen-section analysis. No tracer injection or lymphatic mapping was carried out; the procedure therefore constitutes selective lymph node sampling and not a formal sentinel lymph node biopsy. All four nodes were free of tumor on frozen section. The prominent contralateral level IIa node described on CT showed no suspicious morphological features and was managed by observation, in line with the fact that an elective cervical procedure, if indicated at all, would be confined to the ipsilateral side. No major intraoperative complications were observed. This operation with the robotic system prevented extensive scarring in the orofacial area.

The definitive histopathological examination of the complete resection specimen established the diagnosis of a polymorphous adenocarcinoma of the minor salivary glands, conventional subtype (ICD-O C05.9, 8525/3), which represents a low-grade entity. Immunohistochemistry showed intense positivity for S100 and cytokeratin 7, largely negative staining for p40, and p63 positivity predominantly in the outer areas of the glandular structures. The tumor measured approximately 15 mm at the junction of the hard and soft palate on the left side. After the second resection only small offshoots of up to 2 mm were identified in the subepithelial connective tissue. No viable tumor cells were identified within the adjacent bone. The tumor was resected in sano: The re-resection specimen measured 30 mm in width, from the midline of the palate to the maxillary alveolar crest, 28 mm in length, from the hard to the soft palate, and 15 mm in depth including the bony portions. Furthermore, additional marginal Sects.  (2 to 3 mm) were taken intraoperatively from all margins. All final resection margins were reported as free of tumor. The exact distance between the tumor and the closest final margin, however, can no longer be determined retrospectively from the pathology report or from the available specimens, and this is acknowledged as a limitation of the present report.

There was no lymphovascular invasion (L0 V0), and perineural infiltration could not be unequivocally demonstrated. All four sampled cervical lymph nodes were free of tumor on final histopathological examination (0/4), confirming the intraoperative frozen-section result. The definitive classification according to the current 9th version of UICC/AJCC TNM classification was pT1 pN0 (0/4) cM0, R0.

**Fig. 5:**
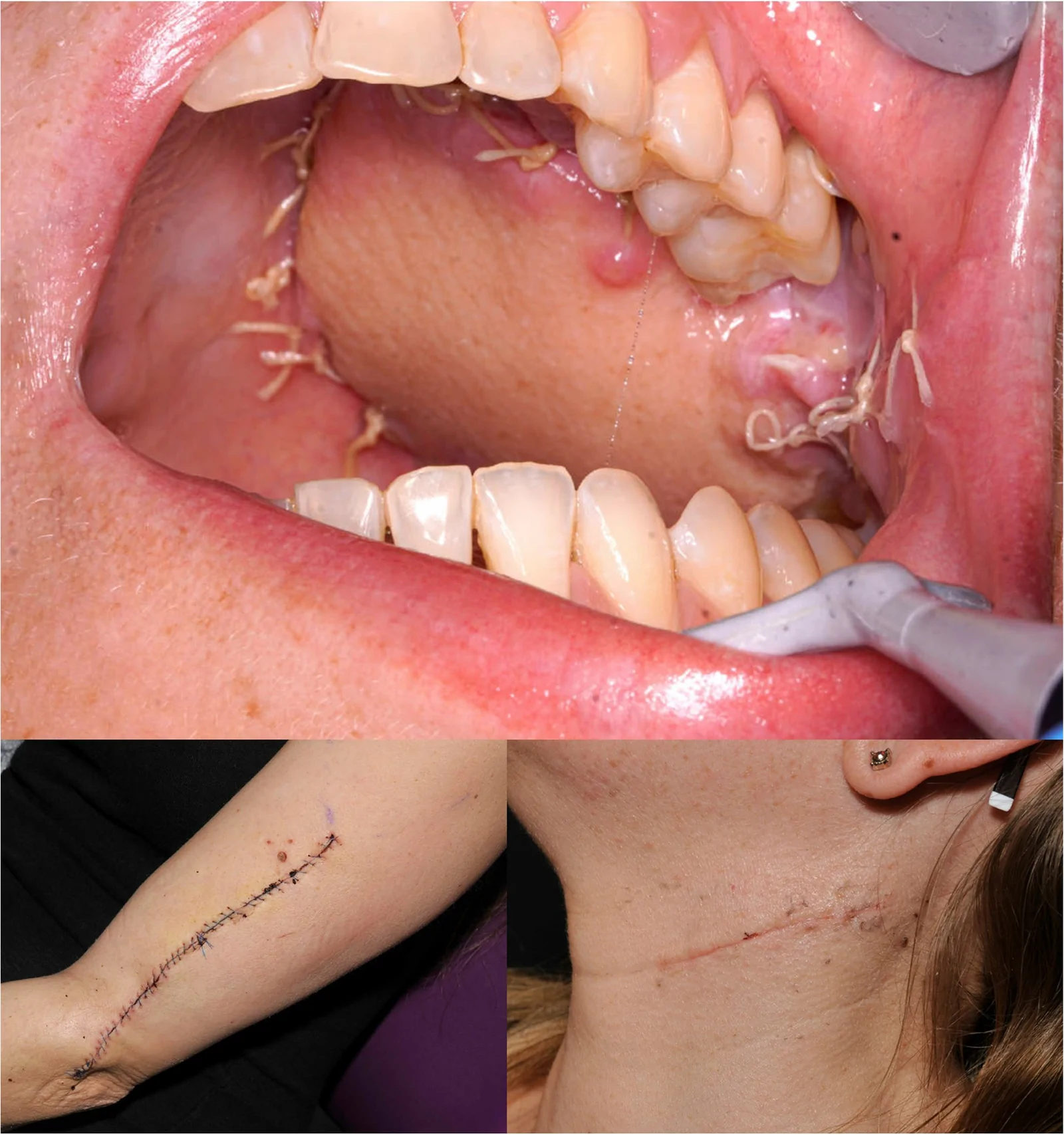
The upper image shows the intraoral graft from the upper arm. The lower images show the extraoral scars from the neck and upper arm

Postoperatively, the patient was monitored in the ICU with flap viability assessments, and early recovery was uneventful. The patient was discharged for outpatient follow-up after two weeks. Side effects included adaptation to the flap, which affected speech, chewing, and swallowing. Long-term physiotherapy and speech therapy were recommended. Overall, the healing process was very successful (Fig. 5). The case was presented to the interdisciplinary tumor board after the definitive histopathological diagnosis was available. Given the low-grade entity, the pT1 stage, the in sano resection and the tumor-free lymph nodes, no adjuvant treatment and no secondary neck dissection were recommended, and regular clinical and sonographic follow-up was agreed upon.

## Discussion and conclusions

Following ablative tumor surgery in the orofacial region, defects can be safely reconstructed using a free graft. Microsurgery and coupler systems enable reliable and predictable results [12]. Nevertheless, there are defects that cannot be reliably treated surgically using conventional microsurgery. For example, coupler systems for veins only extend down to 1 mm. In the sub-1 mm range, we are dealing with supermicrosurgery [13]. In the orofacial region, this is often necessary for reconstructing nerves such as the facial nerve [14].

In this patient case, the vessels had a diameter of just 0.2 mm. Reconstruction via the oral cavity under a microscope appears almost impossible. The 20-fold reduction in motion transmission allowed the vessels to be anastomosed using very fine suture material. Since the free upper arm graft had two arteries, both were connected. The facial artery was thus traced and dissected from the intraoral area to the upper lip. This enabled the two branches of the facial artery to be securely connected. Normally, the anastomosis is performed in the neck, which necessitates significantly larger incisions and results in longer scars. In this way, we achieved a good aesthetic result for the young patient using an alternative surgical method. Performing supermicrosurgery with a robot via the oral cavity is new and represents a new possibility in reconstructive surgery.

In this case, the least invasive approach possible was pursued at every step — an initial biopsy with an unclear tumor entity, transoral resection with a clinical safety margin, re-resection until a histopathologically confirmed safety margin was achieved, and robotic reconstruction — including the management of the cervical lymph nodes. However, cervical lymph node management in malignant salivary gland tumors has to be tailored to entity, grade and tumor stage. In the presence of a positive cervical lymph node finding in pretherapeutic diagnostics, a therapeutic, modified radical neck dissection is indicated. In the clinically node-negative (cN0) neck, an ipsilateral elective lymphadenectomy is recommended for high-grade histologies, carcinomas with high-grade transformation, advanced tumor size (T3/T4) and, for parotid tumors, extraparenchymal growth. For locally advanced adenoid cystic carcinomas (T3, T4), an elective neck dissection should likewise be considered. For T1/T2 low-grade carcinomas, by contrast, a “wait and see” strategy is regarded as acceptable. Occult cervical metastases are found in approximately 20% of salivary gland carcinomas overall, but this figure is strongly driven by high-grade entities and advanced tumor stages [6].

For the conventional variant of PAC, these general considerations are supported by the entity-specific evidence. Regional metastasis is uncommon, and in the population-based cohort of 460 patients an elective neck dissection was performed in only 5.3% of cases, while the 10-year disease-specific survival reached 96.4% [3]. Long-term follow-up data have identified focal papillary growth as being associated with cervical lymph node involvement, and positive or unknown surgical margins as being associated with local recurrence, whereas distant metastasis remained rare [5]. A more aggressive course, including a relevant rate of cervical metastases, is observed mainly in the cribriform variant of minor salivary glands (CAMSG) and in cases with high-grade transformation, which do warrant elective treatment of the neck [4]. Conversely, a selective neck dissection has been described for PAC in the oropharyngeal region, where the risk profile and the need for adequate local exposure may justify a more extensive regional approach [2]. Taken together, the decisive parameters are the histological subtype and grade, the presence of a cribriform or papillary growth pattern, high-grade transformation, and the T stage — none of which can be reliably determined before the complete specimen has been assessed.

In the present case, the primary tumor was a 15 mm low-grade PAC of the palate, staged pT1, resected in sano and without lymphovascular or unequivocal perineural invasion. According to the criteria outlined above, neither the entity nor the tumor stage constituted an indication for an elective neck dissection, and observation of the cN0 neck would have been an acceptable strategy. Given the patient’s young age and the staging findings, and after discussion with the patient, we nevertheless decided to perform a selective removal of the ipsilateral level II lymph nodes.

It must nevertheless be acknowledged that selective lymph node sampling cannot reliably exclude occult cervical metastases. In contrast to a formal sentinel lymph node biopsy, no tracer injection or lymphatic mapping was performed, so that the removed nodes do not necessarily represent the first echelon of lymphatic drainage, and in contrast to a comprehensive neck dissection, only a limited number of nodes was assessed. The designation pN0 must therefore be interpreted in the context of four examined lymph nodes. A further limitation concerns the final resection margin. Although all margins of the re-resection specimen were free of tumor, the exact minimal distance to the closest margin cannot be quantified retrospectively, so that the completeness of the resection can be stated qualitatively but not in millimetres. Given that margin status is one of the strongest predictors of local recurrence in polymorphous adenocarcinoma [2, 4, 5], this reinforces the need for close long-term follow-up. Given the indolent behavior of conventional PAC, we consider this residual uncertainty acceptable, but it mandates close clinical and sonographic follow-up, particularly since recurrences of PAC are known to occur late [4]. Beyond the individual case, this constellation illustrates a more general point: when the definitive tumor entity is not yet established, resection of the primary tumor with adequate margins should not be delayed, whereas the decision on comprehensive cervical treatment is sometimes better deferred until the final histopathological diagnosis is available. This avoids both under- and overtreatment of the neck and allows the least invasive therapy possible, always in consultation with the patient’s wishes. The same principle applies to the reconstruction performed during tumor surgery.

Functional outcomes, including speech, swallowing, and oropharyngeal fluid dynamics, are critical metrics for evaluating the success of reconstruction. Long-term assessment using objective measures of bolus flow, velopharyngeal closure, and patient-reported quality of life is essential to determine the effectiveness of novel reconstructive strategies. Studies on buccinator myomucosal flaps have demonstrated favorable long-term recovery in these parameters [10], and similar evaluation frameworks should be applied to robotic-assisted free flap reconstruction.

This case presented herein demonstrates the feasibility and potential benefits of robotic-assisted transoral reconstruction using an upper arm free flap following posterior palatal PAC resection. While single-case data cannot establish definitive superiority, the successful transoral robotic-assisted reconstruction with an upper arm free flap in this patient highlights the potential for minimally invasive management of posterior palatal defects. This approach may reduce morbidity associated with extraoral incisions, preserve function, and enable precise microvascular reconstruction, highlighting the potential of robotic-assisted techniques for challenging intraoral defects. Further long-term evaluation of functional outcomes, including objective assessment of fluid dynamics, speech, and patient quality of life, will be essential to establish the broader applicability of this approach.

## Data Availability

The datasets used and/or analysed during the current study are available from the corresponding author on reasonable request.
